# Retroperitoneal hematoma with bone resorption around the acetabular component after total hip arthroplasty: a case report and review of the literature

**DOI:** 10.1186/1752-1947-6-294

**Published:** 2012-09-13

**Authors:** Kenzo Uchida, Kohei Negoro, Yasuo Kokubo, Takafumi Yayama, Tsuyoshi Miyazaki, Hideaki Nakajima, Ai Yoshida, Hisatoshi Baba

**Affiliations:** 1 Departments of Orthopaedics and Rehabilitation Medicine, Faculty of Medical Sciences, University of Fukui, Matsuoka-Shimoaizuki 23-3, Eiheiji, Fukui, 910-1193, Japan

**Keywords:** Total hip arthroplasty, Retroperitoneal hematoma, Bone resorption, Revision surgery

## Abstract

**Introduction:**

Vascular complications related to cup-fixating screws penetrating the medial acetabular wall during total hip arthroplasty are not uncommon but rarely are associated with serious adverse events in the late post-operative period.

**Case presentation:**

We present the case of a 77-year-old Japanese woman who developed progressive extensive bone resorption and large hematoma in the acetabulum 13 years after total hip arthroplasty. On admission to our hospital, she was on oral warfarin (1.5mg/day) for atrial fibrillation. About 5 months after the initiation of anticoagulant therapy, she suffered a major fall followed by massive subcutaneous and pelvic girdle bleeding, predominantly on the medial side of the right thigh, but a fracture or damage of total hip arthroplasty was not evident on an emergency orthopedic evaluation. One year after the accident, a routine follow-up examination showed an asymptomatic osteolytic lesion in the acetabulum on the right pelvis, and 2 years later our patient noticed progressive pain in her right hip during walking. A large osteolytic lesion was noted in the right acetabulum on a plain radiograph. On high-resolution computed tomography and magnetic resonance imaging, a huge granulomatous lesion in the acetabulum was suggestive of chronic hematoma in intrapelvic and extrapelvic gluteal regions. A closer computed tomography examination showed that one of the screws used for fixation of the acetabular component in the total hip arthroplasty had penetrated the acetabular bone and had reached the pelvic cavity. Surgery was performed in a single session by means of two approaches: anterior midline transperitoneal address to resect the low-density mass lesion followed by posterolateral acetabular implant re-settlement.

**Conclusions:**

Though rare, total hip arthroplasty-related late vascular complications could be serious and potentially affect the limb and quality of life.

## Introduction

Total hip arthroplasty (THA) is a well-established treatment option for hip joint symptomatic osteoarthritis in elderly people. Although THA results in a significant reduction of hip pain and improved quality of life, serious complications, including loosening and migration of the hip implant, can occur. The THA surgical procedure includes resection of the inner lesion of the osteoarthritic acetabulum followed by settlement of a usually high-density polyethylene cup component that often is fixed with several screws and that is introduced from the outside of the acetabulum to the inner pelvic cavity. An improperly introduced acetabular cup-fixating screw usually results in breakage, loosening, or migration of the implant or a combination of these. However, infrequently, this part of the procedure causes injury to the vascular structures or soft tissues (or both), including muscles (iliac muscle) within the pelvic cavity. As we report here, an extremely rare late-stage complication of cup-fixating screws caused slowly expanding hematoma with resultant acetabular bone resorption and loosening of the acetabular component and required THA revision.

## Case presentation

**Figure 1:**
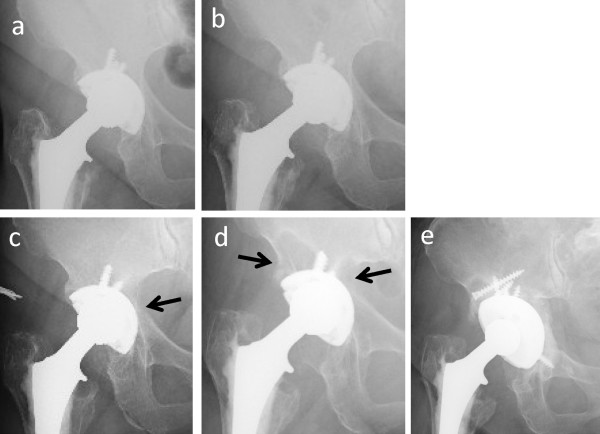
**Serial radiographs of our patient.** (**a**) A radiograph taken immediately after total hip arthroplasty in 1998 shows excellent incorporation of the cementless cup within the acetabulum. (**b**) A radiograph taken immediately after the accidental fall in 2008 shows no obvious fracture or osteolytic lesion. (**c**) A radiograph taken 1 year after the fall shows a small osteolytic lesion (arrow). (**d**) A radiograph on admission in 2010 shows an extended osteolytic lesion in the superolateral side of the ilium and the acetabulum (arrows) and no obvious migration of the cup or screws. (**e**) A post-operative radiograph taken in 2011 after resection of the hematoma lesion and cup revision surgery.

An otherwise previously healthy 77-year-old Japanese woman was admitted to our university medical center with an approximately 4-month history of progressive right hip joint pain. She had a progressive stage of dysplastic hip osteoarthritis on both sides without any other congenital disease, blood disease, or rheumatoid arthritis. At the age of 64, she underwent right-side THA (Natural-Hip™ System #2, APR® cup size of 51mm; Sulzer Medica, Austin, TX, USA) at our institution (Figure 1a). At the age of 73, she underwent left-side THA with a different assembly (S-ROM® hip stem, Pinnacle acetabular cup; DePuy Orthopaedics Inc., Warsaw, IN, USA) at another institution. Soon after the left-side THA, she developed atrial fibrillation and was treated with an anticoagulant (warfarin 1.5mg/day). About 5 months after the initiation of anticoagulant therapy, she suffered a serious fall, causing massive subcutaneous and pelvic girdle hematomas, predominantly on the medial side of the right thigh, but plain radiographs in the emergency orthopedic department showed no fracture, osteolytic lesion, or abnormal findings related to the THA (Figure 1b). One year after the accident, asymptomatic osteolytic lesion in the innominate bone (around the arcuate line) on the right pelvis (Figure 1c) was diagnosed on the basis of the findings of a routine follow-up examination. Two years later (4 months before admission to our university medical center), she complained of pain of a progressive nature in her right hip during walking. Plain radiographs showed a large osteolytic lesion in the right acetabulum without migration of the acetabular cup or screws.

On admission to our hospital, our patient was on oral warfarin (1.5mg/day) for atrial fibrillation but was otherwise in good disease-free condition, apart from the bilateral THA. She had a slightly limited range of movement on her right side, but her hip muscle strength on a manual muscle test was normal bilaterally: Harris hip score was 68 out of 100 points. Palpation of the right iliac and psoas muscles did not elicit any pain. Blood test results were unremarkable: leukocyte count of 5.8×10^9^/L, erythrocyte count of 439×10^9^/L, hemoglobin of 12.7g/dL, hematocrit of 40.4%, platelet count of 181×10^9^/L, prothrombin time of 18.4 seconds, prothrombin activity of 36.3%, activated partial thromboplastin time of 46.0 seconds, and C-reactive protein of 0.12mg/dL. Blood cultures were negative for bacteria and viruses.

**Figure 2:**
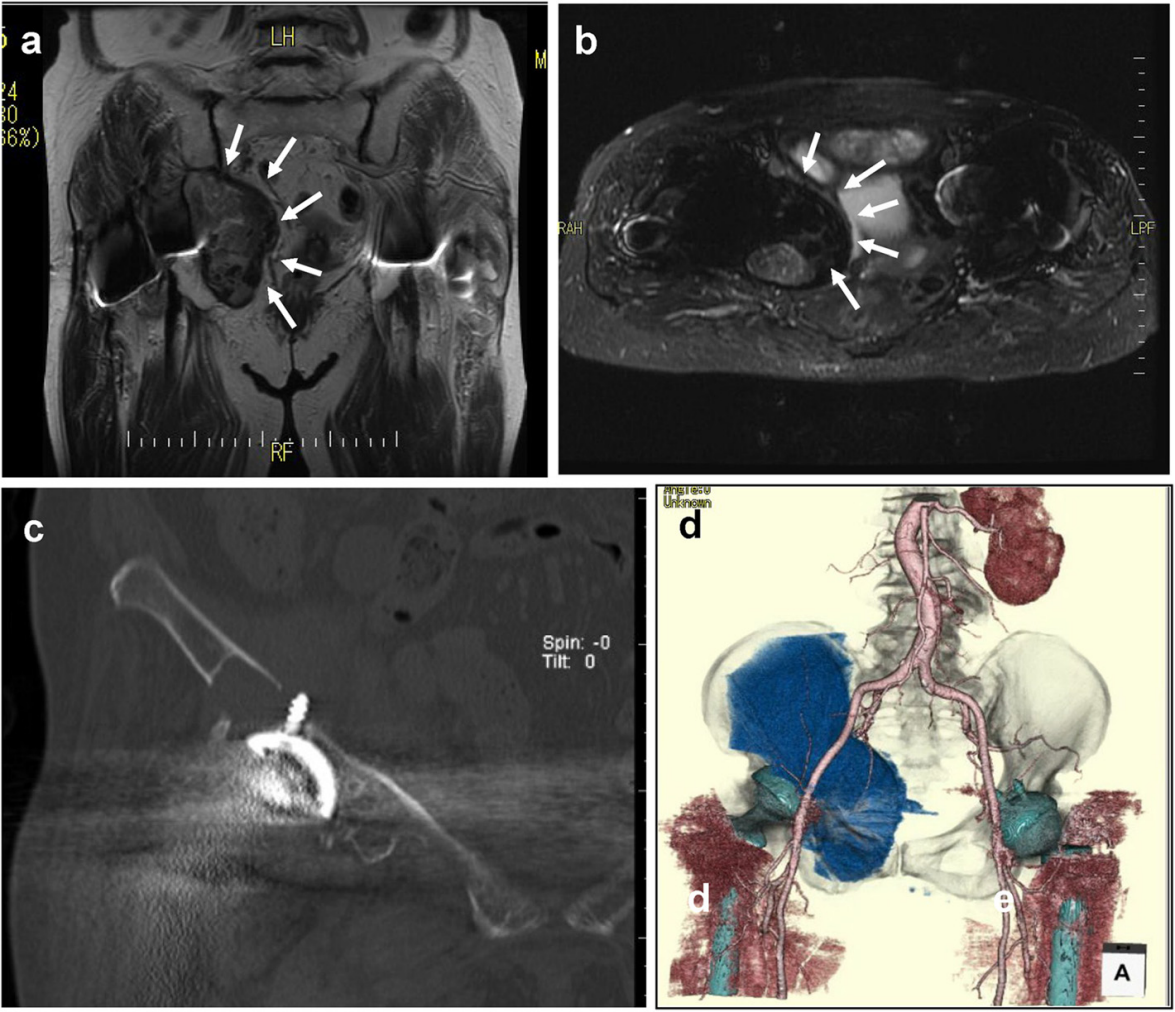
**Imaging findings on admission.** (**a**) A coronal T2-weighted magnetic resonance image (repetition time [TR] of 3800 and echo time [TE] of 74,932) shows a mass of low signal intensity within the pelvis (area surrounded by arrows). (**b**) A short T1 inversion recovery (STIR) image of magnetic resonance imaging (TR of 3700 and TE of 58,786) shows a small area of high density within the low-density lesion (surrounded by arrows). (**c**) High-resolution coronal computed tomography images show penetration of innominate acetabular bone (with partial bone resorption) by the screw tip and a large low-density lesion. (**d**) Three-dimensional magnetic resonance angiography shows a large abnormal lesion that is located between the external and internal artery and vein and that extends widely to the obturator foramen or foramen obturatum.

Plain radiographs taken on admission showed a massive osteolytic lesion (Figure 1d), and magnetic resonance imaging indicated that the lesion was 5.0×6.0cm in size (Figure 2a, b) and neighbored the right iliac bone between the iliac muscle and ilium. The lesion was a mass of low signal intensity and appeared to extend to the extraperitoneal inner pelvic cavity and compress the bladder toward the contralateral side. High-resolution coronal computed tomography (CT) images showed that the screw tip had penetrated the iliac bone from the superior exterior margin of the acetabulum in contiguity to the low-density area while the screw tip remained outside the low-signal-density mass (Figure 2c). The lesion was negative on both 99m-technetium-methylene diphosphonate bone scanning and 67-gallium citrate scintigraphy. Three-dimensional magnetic resonance angiography (GE Healthcare, Milwaukee, WI, USA) demonstrated deviation of the external iliac artery and vein but no link (feeder or drainer) between the low-density mass and the internal iliac artery and vein or their branches (Figure 2d). These findings were highly suggestive of a chronic expanding retroperitoneal hematoma that was located between the iliac and internal obturator muscle, grossly about 5.0×6.0×10.0cm in size, and without contiguity to the major vessels. Warfarin was discontinued and was replaced with heparin (13,000U/day) in preparation for surgery.

**Figure 3:**
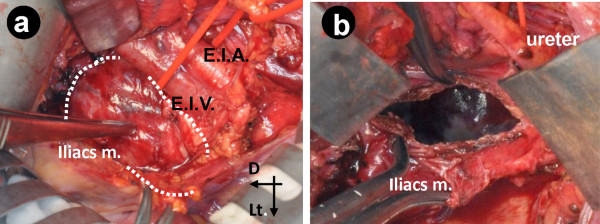
**Intra-operative photographs.** (**a**) After partial dissection of the retroperitoneum followed by protective retraction of the right external iliac artery (E.I.A.) and external iliac vein (E.I.V.), a gross outline of the pseudotumoral lesion (surrounded by dotted curved line) within the iliac muscle is seen. (**b**) Complete drainage of the hematoma, which is shown by the dotted curved line in (a). Part of the acetabular cup through the “vanishing” acetabular (innominate) bone is seen. Arrows show direction: D, distal; Lt., left; Med., medial.

Surgical treatment was planned in a single session at two stages: anterior midline transperitoneal approach (first stage) to resect the low-density lesion followed by posterolateral acetabular implant re-settlement. The anterior transperitoneal midline approach was followed by a retroperitoneal incision to identify the internal and external iliac arteries and veins and their branches. The granulomatous lesion was identified posterolateral to the external iliac artery and vein but without any venous networks or major connection to the vasculature (Figure 3a). The lesion was covered with a dense granular wall that contained a large old hematoma (blood clot) and that widely extended to the area of foramen obturatorium inferiorly and iliac crest superolaterally (Figure 3b). The hematoma was drained completely, and part of the acetabular cup was identified through the empty lesion of the iliac and acetabular bones.

After the left-side decubitus position was changed, the posterolateral address (second stage) was undertaken to explore the acetabular implant. The acetabular cup and three screws were easily removed. The inner cavity of the original acetabulum in the pelvic cavity contained the hematoma-like mass. A massive bone defect was observed at the base of the acetabulum, but the remaining acetabular structure provided enough support for the large cup. The acetabular bone defect was covered by a mesh (hydroxyapatite and poly-L-lactic acid, Super-Fixorb®; DePuy Orthopaedics Inc.) impacted with allogenic morcerized bone and this was followed by settlement of a 60mm inner diameter Trident® acetabular cup and outer liner X3 (Stryker, Kalamazoo, MI, USA). A histological examination revealed minimal inflammatory macrophage infiltration but no metallosis. The post-operative course was uneventful, and our patient resumed daily activities soon after surgery (Figure 1e).

## Discussion

Failure of artificial joint implants is usually associated with improper use or settlement of implants or both, fixation failure with inappropriate fixation with screws, poor bone ingrowth in the hydroxyapatite-coated metal back of the cup or femoral stem, osteopenia in the host bone, infection, and osteolysis with metallosis in the surrounding tissues. Every effort has been made to enhance the early stable fixation with optimal adaptation of implant to the osteoarthritic hip through technical as well as material innovation of the THA assembly. In the acetabulum, anatomical incongruence of the ilium and the innominate bone is often one of the major causes of loose acetabular cup after THA. A shallow acetabulum and thin iliac bone in the dysplastic hip often require multiple-screw fixation, which could potentially increase the risk of penetration of the ilium and injury of the venous networks running interior to the ilium. Wasielewski *et al*. [1] reported that the iliac vessels are at risk of injury when penetration of the inner cortex of the pelvis occurs in the anterosuperior quadrant of the acetabulum. However, the inner wall (cortex) of the acetabulum is anatomically covered by the thick iliac muscles, and so the cup-fixating screws are unlikely to injure the major vessels such as the internal and external iliac vessels, with the exception of the superior artery and vein. Any muscle trauma that might occur with this technical error is usually insignificant but, in conjunction with the use of anticoagulant therapy, may cause sufficient bleeding to form an iliac hematoma [2]. Hematomas are usually reabsorbed and decrease in size slowly but, in rare cases, can slowly increase in size. Such hematomas include pseudotumors, such as those seen in patients with hemophilia and other disorders. Reid *et al*. [3] designated such a lesion in non-hemophiliacs as chronic expanding hematoma, which is defined as a hematoma that is caused by trauma or other etiology, is located on the muscle fascia or between muscles, does not show complete absorption, undergoes necrotic degradation and liquefaction, becomes cystic and forms a foreign body granuloma, and is encased in a fibrous capsule.

In our patient, obvious fracture (although microfracture could not be excluded) or migration of the cup or screws was not evident on the plain radiographs taken immediately after the fall or at follow-up, in spite of the expansion of the ilium and acetabulum. In contrast, CT images before THA revision showed penetration of the inner cortex of the acetabulum. The tip of the acetabular cup-fixating screw could have penetrated the inner cortex on the left side of the primary THA at the age of 64, although such a technical error was considered insignificant at the time. The accidental fall probably resulted in injury of the microvessels and venous networks in the soft tissues, including the periosteum or iliac muscles, by the screw tip without obvious fracture evident on the plain radiographs. A slowly growing expansive hematoma associated with anticoagulant therapy is likely to have developed during the subsequent 2-year period, both inside and outside the iliac bone, and to have resulted in resorption of the acetabular bone and loosening of the acetabular component. The screw tip in the actively contracting iliac muscle could have continued to cause a progressive increase in the size of the hematoma. The bony changes and destruction that appeared as osteolytic changes could also be the results of recurrent bleeding, as seen in patients with hemophilia [4]. Due to the slow debris formation, minimal wear, and large blood volume in the lesion, particle-induced osteolysis seems less likely [5]. Local instability also caused bone resorption in the superolateral area of the acetabulum. This probably contributed to the instability of the local implant assembly and pain on walking.

Though rare, THA-related vascular complications are often serious and potentially affect the limb and quality of life. Previous studies have described THA-related iliac or retroperitoneal hematoma [2,6-10] (Table 1). Among these six cases, four were discovered in the early post-operative period and the remaining two were discovered after at least 6 months. All four cases of early onset had femoral nerve paralysis complications. No neurologic symptoms were observed in cases discovered 3 years or more after surgery. Other than femoral nerve paralysis, the main featured symptoms were hip joint pain and the progression of anemia. Anticoagulant therapy, including therapy as post-operative prevention against deep vein thrombosis, was also carried out in four cases. In our case of chronic onset, no femoral nerve paralysis was observed, and hip joint pain was the initial symptom. However, anticoagulant therapy was carried out, and anemia may have worsened with the progressive increase in the size of the subcutaneous hematoma around the hip joint at the time of the fall. In cases in which these symptoms are present after surgery, the possibility of retroperitoneal hematoma should be considered.

**Table 1:** Summary of previous case reports of retroperitoneal hematoma after total hip arthroplasty

**Reference**	**Age, years**	**Sex**	**Site**	**Size, cm**	**Morbidity span**	**Symptoms**	**Anticoagulant therapy**	**Treatment**
[2]	61	Female	Right	NA	8 days	Femoral nerve palsy	+	Conservative
[6]	65	Male	Left	10×11×15	2 days	Femoral nerve palsy, anemia	−	Excision/screw removal
[7]	78	Female	Right	16×11	10 days	Bilateral hip and thigh pain, anemia	+	Conservative
[8]	67	Female	Left	9×7.5×7	9 days	Femoral nerve palsy, anemia	+	Conservative
[9]	76	Female	Right	4×6	6 months	Femoral nerve palsy	−	Excision/transcatheter arterial embolism
[10]	71	Male	Right	NA	3 years	Hip joint pain	+	Excision/cup revision

*NA* not available.

## Conclusions

In the case described here, an extremely rare possibly late-stage complication of cup-fixating screws in THA caused progressive development of chronic expanding hematoma, resultant acetabular bone resorption, and loose acetabular component and required revision THA. Owing to the continued need for anticoagulant therapy in such cases, meticulous attention must be paid to avoid trauma that could produce a new hemorrhage around the THA implant.

## Abbreviations

CT, computed tomography; THA, total hip arthroplasty.

## Competing interests

The authors declare that they have no competing interests.

## Authors’ contributions

KU drafted the manuscript. YK was the main surgeon in charge. KN, TM, and AY were part of the surgical team and were responsible for the post-operative care. TY and HN revised the manuscript and provided clinical support. HB participated in the conceptualization and final version of the manuscript. All authors read and approved the final manuscript.

## Consent

Written informed consent was obtained from the patient for publication of this case report and accompanying images. A copy of the written consent is available for review by the Editor-in-Chief of this journal.
